# Overexpression of a cell wall damage induced transcription factor, OsWRKY42, leads to enhanced callose deposition and tolerance to salt stress but does not enhance tolerance to bacterial infection

**DOI:** 10.1186/s12870-018-1391-5

**Published:** 2018-09-03

**Authors:** Shakuntala E. Pillai, Chandan Kumar, Hitendra K. Patel, Ramesh V. Sonti

**Affiliations:** 1 CSIR-Centre for Cellular and Molecular Biology, Uppal Road, Hyderabad, 500007 India; 2 National Institute of Plant Genome Research, New Delhi, 110067 India

**Keywords:** Cell wall degrading enzymes, WRKY transcription factor, *OsWRKY42*, Callose deposition, Salinity stress, Jasmonic acid

## Abstract

**Background:**

Members of the WRKY gene family play important roles in regulating plant responses to abiotic and biotic stresses. Treatment with either one of the two different cell wall degrading enzymes (CWDEs), LipaseA and CellulaseA, induces immune responses and enhances the expression of *OsWRKY42* in rice. However, the role of OsWRKY42 in CWDE induced immune responses is not known.

**Results:**

Expression of the rice transcription factor *OsWRKY42* is induced upon treatment of rice leaves with CWDEs, wounding and salt. Overexpression of *OsWRKY42* leads to enhanced callose deposition in rice and Arabidopsis but this does not enhance tolerance to bacterial infection. Upon treatment with NaCl, Arabidopsis transgenic plants expressing *OsWRKY42* exhibited high levels of anthocyanin and displayed enhanced tolerance to salt stress. Treatment with either cellulase or salt induced the expression of several genes involved in JA biosynthesis and response in Arabidopsis. Ectopic expression of *OsWRKY42* results in reduced expression of cell wall damage and salt stress induced jasmonic acid biosynthesis and response genes. *OsWRKY42* expressing Arabidopsis lines exhibited enhanced tolerance to methyl jasmonate mediated growth inhibition.

**Conclusion:**

The results presented here suggest that *OsWRKY42* regulates plant responses to either cell wall damage or salinity stress by acting as a negative regulator of jasmonic acid mediated responses.

**Electronic supplementary material:**

The online version of this article (10.1186/s12870-018-1391-5) contains supplementary material, which is available to authorized users.

## Background

The plant cell wall plays a crucial role in development and in adaptation to abiotic and biotic stresses [1]. As a part of their virulence repertoire, plant pathogens produce an array of cell wall degrading enzymes (CWDEs) to degrade different components of the cell wall. The action of CWDEs releases cell wall degradation products, which are categorized as damage-associated molecular patterns (DAMPs) [1–3]. DAMPs act as elicitors of plant immune responses [4]. The perception of DAMPs by plants induces a cascade of signalling events which activate immune responses including expression of *Pathogenesis Related (PR)* genes, production of reactive oxygen species (ROS), lignin deposition and hypersensitive response (HR) [5]. These responses can provide enhanced tolerance to subsequent infection by pathogens [3–6] and this is termed as DAMP triggered immunity (DTI). Treatment with cell wall derived products like oligogalacturonides (OGs), low concentration of hepta-β-glucoside and oligoxyloglucans also elicit DTI responses [5, 7]. Cellobiose, a degradation product of cellulose, also serves as a DAMP and induces defense-like responses [8]. The molecular players involved in DTI are not well characterised. We are trying to understand the mechanisms involved in CWDE induced DTI in *Oryza sativa* (rice).

*Xanthomonas oryzae* pv. *oryzae* (*Xoo*), the causal agent of bacterial blight disease in rice secretes several plant cell wall degrading enzymes as part of its virulence repertoire. Treatment of rice tissues with any of the *Xoo* secreted CWDEs such as cellulaseA (ClsA), cellobiosidase (CbsA)and lipaseA/esterase (LipA) induces plant immune responses such as callose deposition and also primes the plant for defense against subsequent *Xoo* infection [6]. Transcriptome analysis of rice leaves shows that many defense response associated genes are upregulated upon treatment with either LipA or ClsA and that the jasmonic acid (JA) mediated defense response may also be upregulated under these conditions [9, 10]. In addition, levels of JA-Isoleucine were found to be elevated upon treatment with LipA. A significant proportion of the differentially expressed genes are transcription factors. Approximately 17% (26 /152 genes) and 9% (68/720 genes) of the total upregulated genes upon treatment with either ClsA or LipA, respectively, are transcription factors [9, 10]. This suggests that transcription factors may be playing a vital role in the elaboration of CWDE induced immune responses in rice. The WRKY family of transcription factors act as positive as well as negative regulators of biotic and abiotic stress responses [11]. We identified a WRKY transcription factor, *OsWRKY42*, whose expression is induced upon treatment with various CWDEs. Microarray analysis indicated that expression of *OsWRKY42* was induced in rice leaves 12 h post treatment with either LipA or ClsA [9, 10]. Microarray analysis also indicated that expression of this gene is upregulated even at 2 h post treatment with LipA (A Ranjan and R V Sonti, unpublished data; GSE53940). Interestingly, this was the only transcription factor whose expression was found to be induced in early (2 h) and late (12 h) time points after treatment with LipA. Recent studies have also shown that *OsWRKY42* expression is induced as early as one hour after *Magnaporthe oryzae* infection in japonica rice cultivar Mudanjiang 8*.* Overexpression of *OsWRKY42* enhanced susceptibility to *M. oryzae* infection in rice by suppression of jasmonic acid responses [12].

In the present study, we have tried to understand the possible role of *OsWRKY42* in the elaboration of DTI. Our findings demonstrate that overexpression of *OsWRKY42* leads to enhanced callose deposition, a defense response, but does not confer enhanced tolerance to bacterial pathogens in either rice or Arabidopsis. Heterologous expression of *OsWRKY42* in Arabidopsis leads to enhanced salt stress tolerance. *OsWRKY42* expression suppresses cellulase and salt stress induced expression of jasmonic acid biosynthesis and responsive genes in Arabidopsis. Our results suggest that the role of *OsWRKY42* in DTI might be to dampen JA responses that are induced following cell wall damage.

## Results

### Expression of *OsWRKY42* is induced upon treatment with CWDEs

**Fig. 1:**
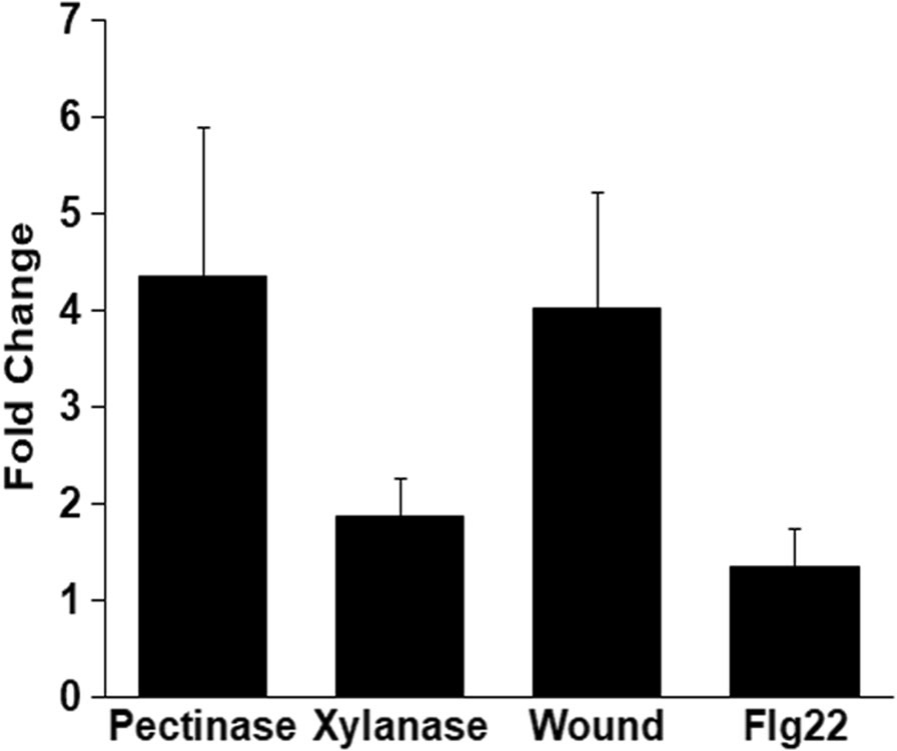
Expression of *OsWRKY42* is induced by either wounding or treatment with cell wall degrading enzymes but not upon treatment with Flg22. Leaves of fourteen days old rice seedlings were syringe infiltrated with any one of the following: water (control), pectinase (2 U/ml), xylanase (2 U/ml), Flg22 (100 μM). For wounding, leaves (*n* = 15) were punctured 8–10 times using a needle (0.45 × 13 mm). Twelve hours post treatment, leaves were harvested and processed for qRT-PCR. The relative fold change was calculated over control (for infiltration with cell wall degrading enzyme or flagellin) or untreated leaves (for wound treatment). *OsGAPDH* was used as endogenous control. Graph represents the mean from three biological replicates and error bar represents standard deviation

Transcriptional profiling of rice leaves that were treated with either LipA or ClsA indicated that the expression of *OsWRKY42* was upregulated at 12 h post treatment [9, 10]. *OsWRKY42* expression was also found to be induced in rice leaves at 2 h after treatment with LipA (A Ranjan and R V Sonti, unpublished data; GSE53940). Interestingly, *OsWRKY42* was the only transcription factor whose expression was upregulated at both 2 h and 12 h post LipA treatment. Rice leaves were also treated with commercially available CWDEs like pectinase or xylanase in order to determine if the expression of *OsWRKY42* is induced following treatment with other CWDEs. The expression of *OsWRKY42* was found to be induced by 4-fold after pectinase treatment and 1.5 to 2-fold after treatment with xylanase (Fig. 1). This is comparable to the levels obtained after treatment with either ClsA or LipA in previously performed microarray analysis [9, 10]. Expression of the *OsWRKY42* gene was also elevated by four-fold after wounding. However, *OsWRKY42* expression was not induced after treatment with Flg22 (Fig. 1). Similar results were obtained in three independent experiments.

### Overexpression of *OsWRKY42* leads to enhanced callose deposition but does not provide enhanced tolerance to *Xoo* infection in rice

**Fig. 2:**
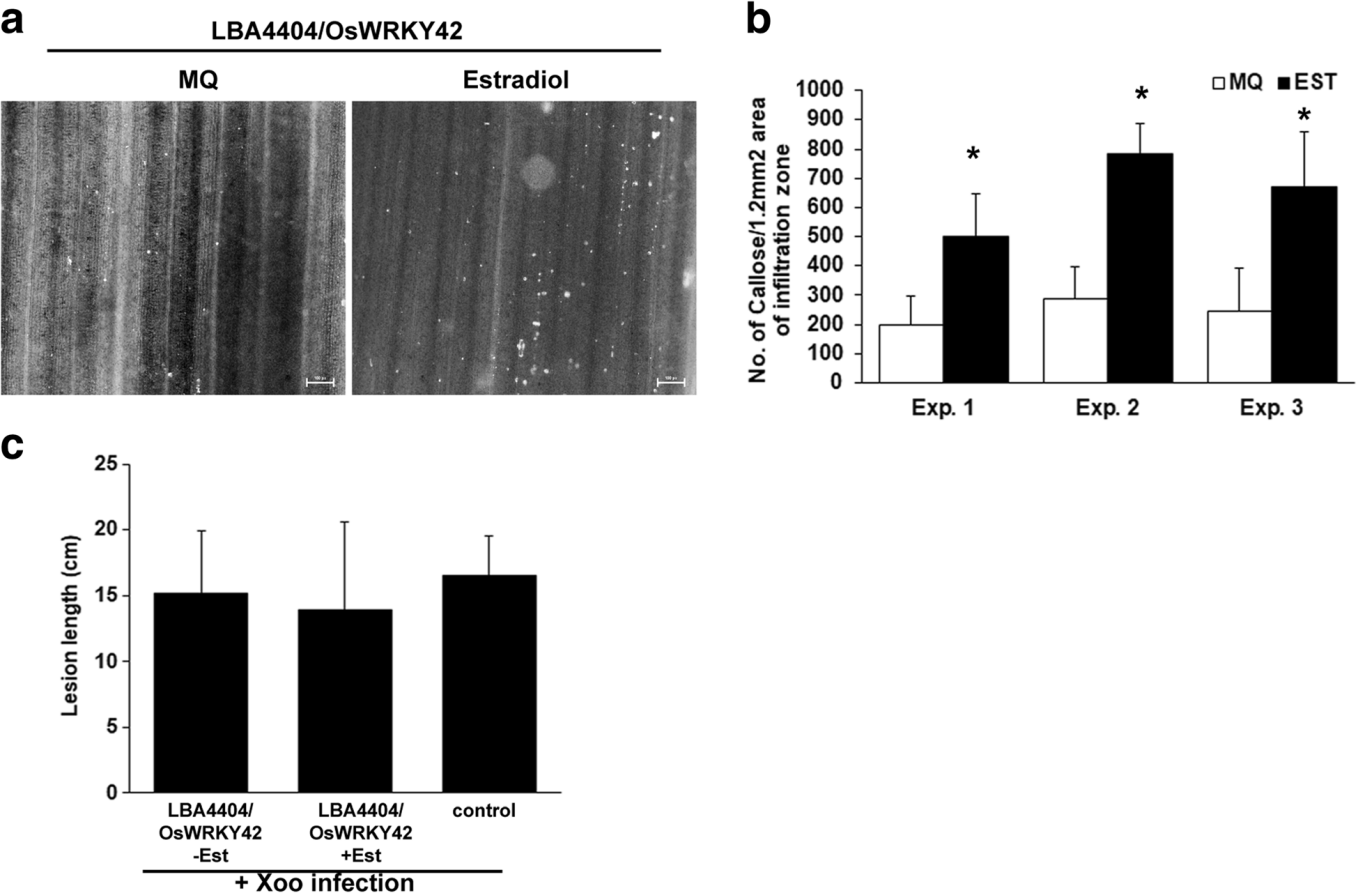
Transient overexpression of *OsWRKY42* enhances callose deposition but does not provide enhanced tolerance to bacterial infection in rice leaves. For assaying callose deposition, the leaves (*n* = 10) of fourteen days old TN-1 rice seedlings were syringe infiltrated with *Agrobacterium* strain LBA4404/pMDC7::OsWRKY42 along with either 20 μM 17-β Estradiol (Est) or water. **a.** After 16 h, the leaves were collected, stained with aniline blue and visualized under an epifluorescence microscope. Callose deposits are seen as white spots in the images. The scale bar represents 50 μm. **b**. The number of callose deposits per area of view was counted manually and the average of ten leaves was plotted. **c**. *Xoo* infections were carried out in mid veins of leaves (*n* = 15–20) of 40 days old TN-1 rice plants. The midveins were preinjected with LBA4404/pMDC7::OsWRKY42 with or without estradiol. After 12 h, inoculation with *Xoo* was performed 1–2 cm below the point of Agrobacterial injection by pricking with a needle dipped in a saturated culture of *Xoo*. The lesion was seen as yellowing of the midvein and the lesion length was measured 10 days post infection. The bar represents average lesion length. Error bar represents standard deviation. Data was analysed using the Student’s *t*-test for independent means (*indicates significant difference with *p* value < 0.05). Similar results were obtained in four independent experiments

Callose deposition is a hallmark of the plant immune response. It not only acts as a physical barrier but also possesses an array of antimicrobial compounds that are impregnated in it [13]. *OsWRKY42* was cloned in the 17-β estradiol inducible vector, pMDC7. *OsWRKY42* was transiently overexpressed in rice leaves by infiltrating agrobacterial strain LB4404/pMDC7::OsWRKY42 either with or without 17-β estradiol. 17-β estradiol alone does not induce callose deposition in rice leaves [14]. In qPCR analysis the expression of *OsWRKY42* was elevated by 2 to 3-fold, 12 h after infiltration (data not shown). Transient overexpression of OsWRKY42-2XFLAG protein was confirmed by Western blotting (Additional file 1: Figure S1). Rice leaves overexpressing *OsWRKY42* exhibited two to three-fold higher number of callose deposits as compared to the control (*p* < 0.05) (Fig. 2a-b).

As the overexpression of *OsWRKY42* induced callose deposition, we assessed the possibility that it might provide enhanced tolerance to *Xoo* (BXO43 strain) infection in rice. For this purpose, *OsWRKY42* was transiently expressed in the midveins of rice leaves and were subsequently infected by pricking with a needle dipped in a culture of *Xoo*. Disease progression was measured in the form of lesion lengths on the tenth day post infection. In the presence and absence of the inducer, the lesion lengths (approximately 15 ± 5 cm long) were similar with no significant differences (Fig. 2c). The rice leaves that had been infected only with *Xoo*, without any prior treatment with LBA4404, also exhibited lesions of a similar size (approximately 16 cm long). Thus, overexpression of *OsWRKY42* enhanced callose deposition but did not provide enhanced tolerance to *Xoo* infection in rice.

### Ectopic expression of *OsWRKY42* leads to enhanced callose deposition but does not provide enhanced tolerance to bacterial infection in Arabidopsis

**Fig. 3:**
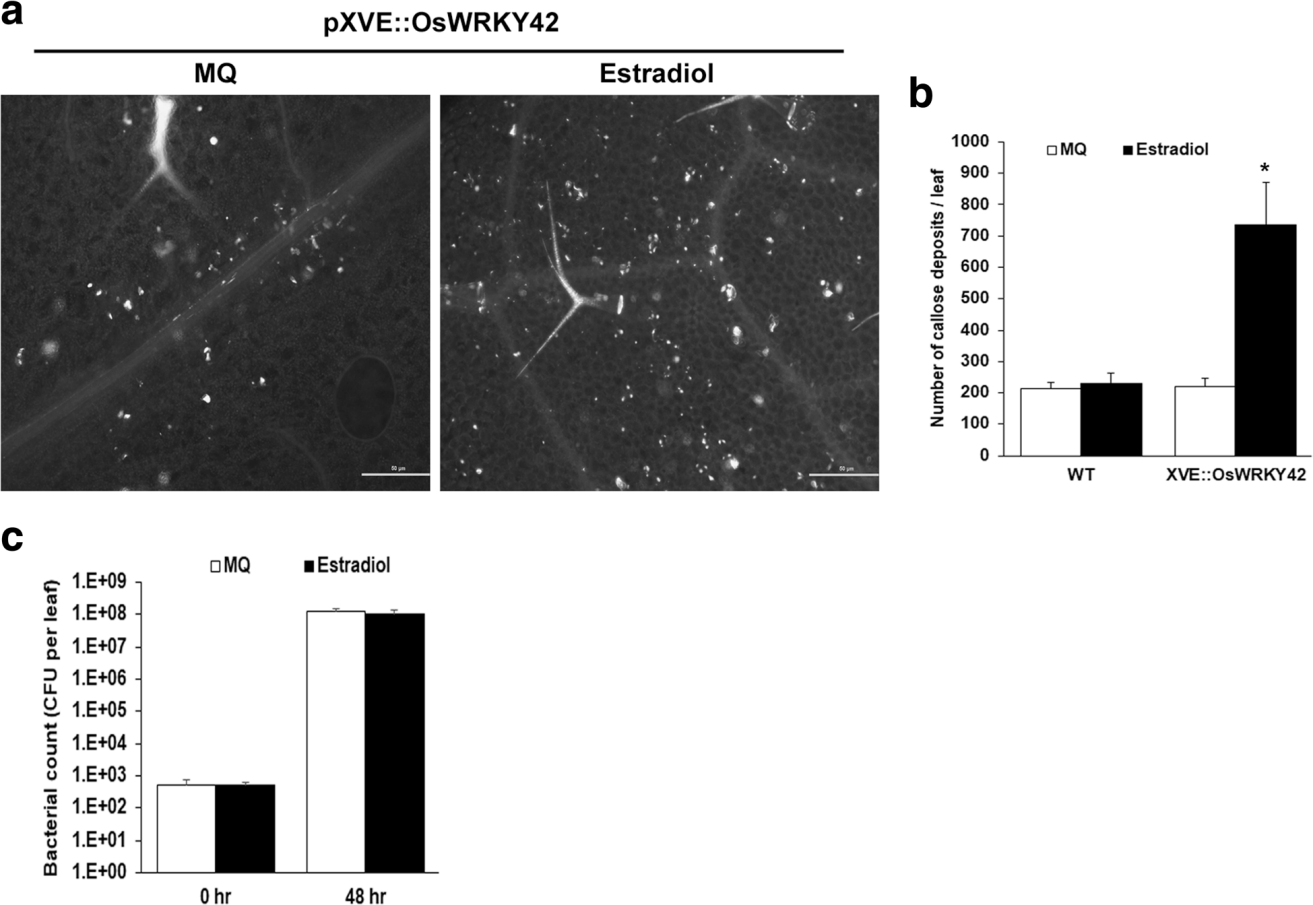
Ectopic expression of *OsWRKY42* in Arabidopsis induces callose deposition but does not provide enhanced tolerance to bacterial infection. Leaves of three weeks old transgenic Arabidopsis plants of T_2_ generation carrying 17 β-estradiol inducible *OsWRKY42* (XVE::OsWRKY42) were infiltrated either with inducer (20 μM 17-β-estradiol) or water using a 1 ml needleless syringe. **a.** For assaying callose deposition, leaves were harvested after 16 h, stained with aniline blue and observed under an epifluorescence microscope. The white spots represent callose deposits. The scale bars in the images represent 50 μm. **b**. The number of callose deposits per leaf were counted manually. The graph represents the average number of callose deposits per leaf (*n* = 5–6). **c**. For *Pst* infection assays, estradiol pre-infiltrated leaves were infiltrated with cells of a *Pst* culture (OD = 0.01). Samples (3 leaves per plant) were collected, 0–2 days post infection (dpi) and processed for estimating total bacterial counts. The number of colony forming units (CFU)/leaf was calculated in leaves from five independent plants each for induced (estradiol) and uninduced (water) sets. Data was analysed using the Student’s *t*-test for independent means (*indicates significant difference with *p* value < 0.05). Error bars represent standard deviation. All of the above experiments were repeated in three independent XVE::OsWRKY42 transgenic lines

Transgenic Arabidopsis lines expressing *OsWRKY42* under the control of an estradiol inducible promoter (XVE::OsWRKY42) were generated. The effect of *OsWRKY42* expression on callose deposition was studied in three independent lines of T_2_ generation of XVE::OsWRKY42 plants that expressed high levels (30–75-fold) of *OsWRKY42* after estradiol treatment (Additional file 2: Figure S2). Rosette stage leaves of wild type and XVE::OsWRKY42 plants, infiltrated with either estradiol or water, were harvested at 16 h post treatment and further processed for callose staining. Induction of *OsWRKY42* expression led to a three to five-fold increase in the number of callose deposits (Fig. 3a-b). The number of callose deposits after estradiol infiltration in wild type plants were comparable to the number of callose deposits in water infiltrated leaves indicating that estradiol does not induce callose deposition in wild type plants (Fig. 3a-b).

In order to determine whether *OsWRKY42* expression would provide enhanced tolerance to bacterial infection in Arabidopsis, transgenic lines expressing *OsWRKY42* were infected with *Pseudomonas syringae* pv. *tomato* (*Pst)* in the presence and absence of estradiol. For these assays, bacteria were infiltrated in rosette stage leaves of adult plants. Two days post infection, the average bacterial counts were 9 × 10^8^ and 12 × 10^8^ colony forming units (CFU) per leaf with and without the expression of *OsWRKY42*, respectively. There was no significant difference observed in disease progression in the presence or absence of *OsWRKY42* expression even after four days post-infection (data not shown). Thus, ectopic expression of *OsWRKY42* did not enhance tolerance to bacterial infection in Arabidopsis (Fig. 3c).

### Expression of *OsWRKY42* is upregulated during salt stress in rice

**Fig. 4:**
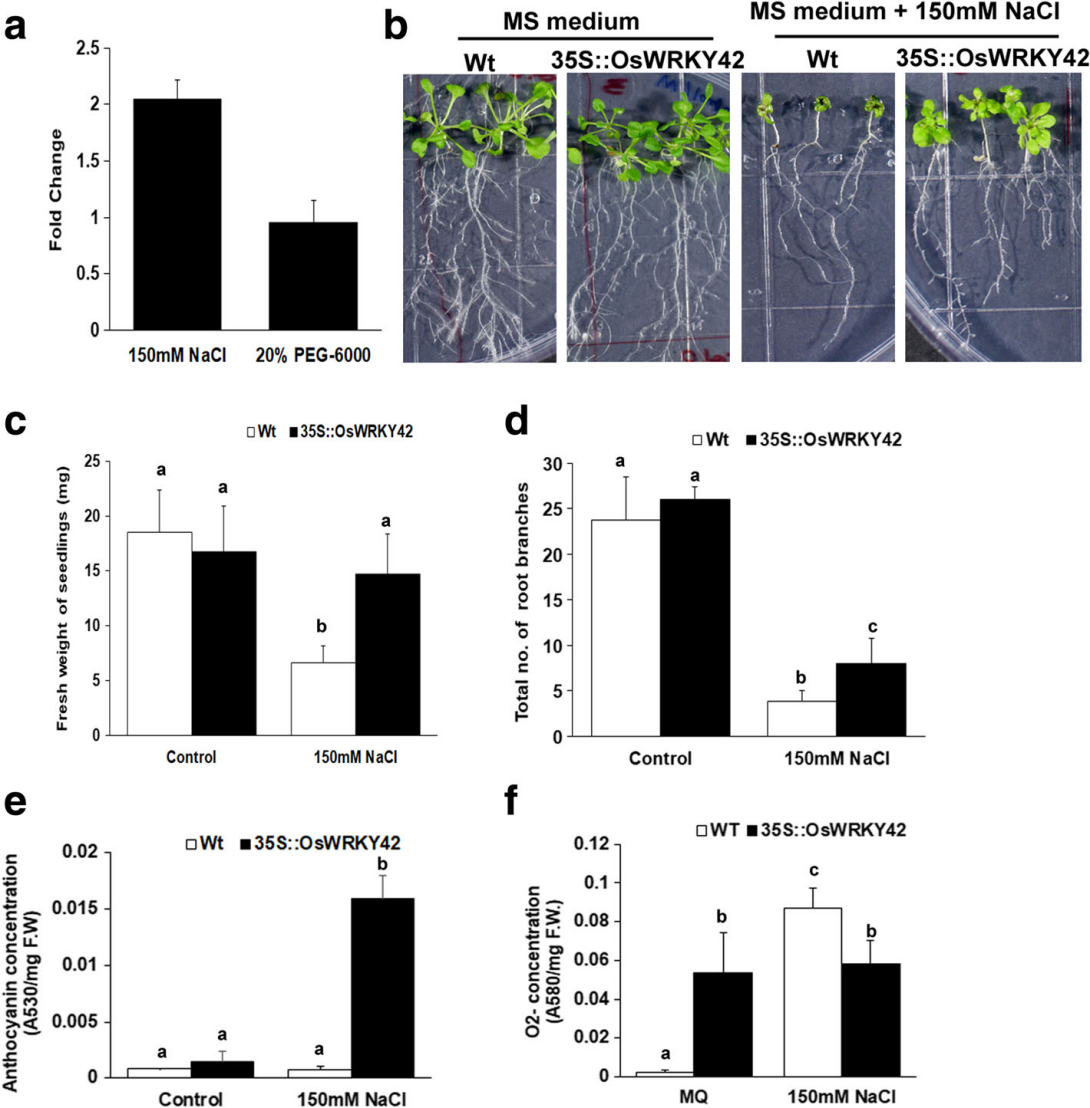
Ectopic expression of *OsWRKY42* enhances tolerance to salt stress in Arabidopsis transgenic lines. **a.** One-week-old TN-1 rice seedlings (n = 10) were dipped in each of the following: 150 mM NaCl solution, 20% (*w*/*v*) PEG-6000 and mock (water). Leaves were harvested 12 h post treatment and processed for qRT-PCR. Relative fold change was calculated over water treated samples and *OsGAPDH* was used as an internal control. **b.** For assaying salt tolerance, one-week-old Arabidopsis seedlings that are either constitutively expressing *OsWRKY42* (35S::OsWRKY42) or wild type (Col-0) (*n* = 20) were grown on MS agar medium with or without NaCl (150 mM, Merck). **(c-d)** The total fresh weight and number of root branches of individual seedlings was quantified on the fifteenth day post-treatment. **e**. For anthocyanin estimation, three weeks old Arabidopsis plants (wild type and 35S::OsWRKY42) were treated with either 150 mM NaCl or water. On the fifteenth day post treatment, leaves (*n* = 3) were collected and anthocyanin was extracted in acidic methanol. Anthocyanin was estimated using a spectrophotometer and the anthocyanin content is expressed as absorbance per milligram of fresh weight (F.W). **f**. ROS estimation was done in detached leaves from adult plants. Leaf discs (n = 15) of either wild type or 35S::OsWRKY42 lines were placed on sterile filter papers soaked in MS solution either with or without NaCl (150 mM). 12 h post treatment, the leaves were stained with NBT. The O_2_^−^ content was estimated spectrophotometrically and is expressed as change in absorbance per milligram of fresh weight. Leaves from three different plants were used as replicates. The data was analysed using one-way ANOVA and the Tukey-Karmer honestly significance difference test. The alphabets above the bar indicates significant difference with *p* value < 0.05. Presence of the same alphabet above the bar indicates that no significant difference was observed. The experiments were repeated in three independent 35S::OsWRKY42 transgenic lines

Analysis of publicly available microarray datasets revealed that *OsWRKY42* expression is induced in rice following abiotic stresses like drought and salinity (Additional file 3: Table S1). We assessed the levels of *OsWRKY42* expression after treatment with either 150 mM NaCl (as salt stress) or 20% PEG-6000 (as drought stress) in rice seedlings. The expression of *OsWRKY42* was upregulated by two-fold as compared to water treated controls,12 h after NaCl treatment but there was no significant change in expression levels after PEG-6000 treatment (Fig. 4a).

### Ectopic expression of *OsWRKY42* leads to enhanced tolerance to salt stress in Arabidopsis transgenic plants

Transgenic Arabidopsis plants that constitutively express the *OsWRKY42* gene (35S::OsWRKY42) were generated. Three independent 35S::OsWRKY42 lines of T_2_ generation that showed the highest expression (150–300 fold) of *OsWRKY42* in qPCR analysis were used for all experiments. The stable expression of OsWRKY42-GFP protein was confirmed by fluorescence microscopy in root cells of the transgenic Arabidopsis lines (Additional file 4: Figure S3). One-week-old seedlings of wild type and 35S::OsWRKY42 Arabidopsis plants were grown on MS agar medium for fifteen days either with or without 150 mM NaCl. Salt stress leads to reduced growth of both wild type and 35S::OsWRKY42 lines (Fig. 4b-d). However, the growth of wild type seedlings was reduced much more as compared to the lines expressing *OsWRKY42*. In wild type plants, the leaves of seedlings were small in size and root growth was poor as compared to the 35S::OsWRKY42 lines. In the presence of NaCl, the average weight of *OsWRKY42* transgenic lines (measured as total fresh weight) is significantly more than wild type plants (*p* < 0.05) (Fig. 4c). Salt mediated suppression of root growth was observed in both wild type and 35S::OsWRKY42 lines. However, the wild type seedlings had significantly lesser number of root branches as compared to 35S::OsWRKY42 transgenic lines (Fig. 4d). Total fresh weight and root branching of wild type and 35S::OsWRKY42 were comparable in absence of salt (Fig. 4c-d). Similar results were obtained with three independent transgenic lines in three experimental replicates.

### Ectopic expression of *OsWRKY42* induces anthocyanin production in response to salt stress

Three weeks old wild type and 35S::OsWRKY42 plants were treated with either 150 mM salt or water for fifteen days. After salt treatment, the 35S::OsWRKY42 lines produced significantly (*p* < 0.05) higher amounts of anthocyanin (Fig. 4e). The wild type plants displayed more wilting as compared to 35S::OsWRKY42 lines (data not shown). Salt stress is always associated with increase in ROS production [15]. Therefore, we estimated levels of the oxygen radical (O_2_^−^) using nitro blue tetrazolium (NBT). Leaves of three weeks old wild type and 35S::OsWRKY42 Arabidopsis were treated with either NaCl (150 mM) or water for 12 h. Salt treatment induced ROS in wild type by 30-fold (Fig. 4e). The basal level of ROS in 35S::OsWRKY42 plants was significantly higher than the untreated wild type. Interestingly, the level of ROS did not increase further after salt treatment in 35S::OsWRKY42 transgenic lines (Fig. 4e).

### Ectopic expression of *OsWRKY42* results in suppression of salt and cellulase induced expression of JA biosynthesis and responsive genes in Arabidopsis

**Fig. 5:**
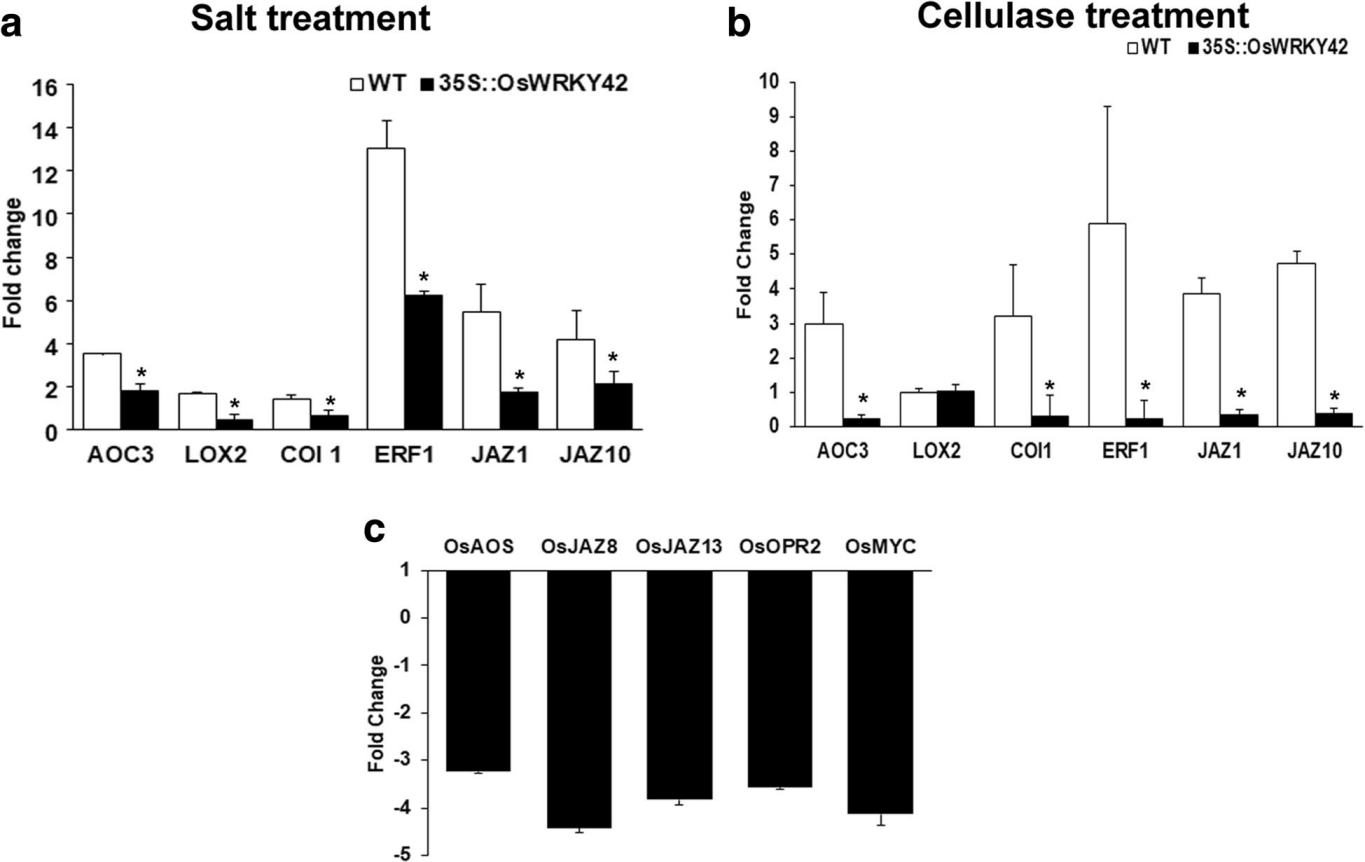
Ectopic expression of *OsWRKY42* suppresses salt and cellulase induced expression of JA biosynthesis and responsive genes in Arabidopsis transgenic lines. Expression of JA biosynthesis and response genes was measured in 35S::OsWRKY42 transgenic lines and wild type (Col-0) Arabidopsis plants after salt and cellulase treatment. **a.** For salt treatment, one-week old seedlings (wild type or 35S::OsWRKY42) were treated for 12 h with either MS or MS with 150 mM NaCl. **b.** For cellulase treatment, leaves of three weeks old Arabidopsis plants (wild type or 35S::OsWRKY42) were infiltrated with 2 U/ml of cellulase (Sigma)*.* Leaves were harvested after 12 h and processed for qPCR analysis. The graph represents the relative fold change using expression values from wild type or 35S::OsWRKY42 lines treated with water. **c.** Leaves of fourteen days old rice seedlings were syringe infiltrated with either LBA4404 or LBA4404/pH7FWG2::OsWRKY42. Twelve hours post treatment, leaves were harvested and processed for qRT-PCR. The relative fold change was calculated over control (leaves infiltrated only with LBA4404). *AtUBQ5* and *OsGAPDH* were used as endogenous controls for rice and Arabidopsis, respectively. The average from three biological replicates is plotted on the graph. The error bar represents standard deviation. Data was analysed using the Student’s *t*-test for independent means. The asterisk above the bar indicates significant difference with *p* value < 0.05. The Arabidopsis experiments were repeated in three independent 35S::OsWRKY42 transgenic lines

Microarray analysis of rice leaves treated with either ClsA or LipA suggested that the JA dependent response pathway is induced upon cell wall damage [9, 10] . A recent study also shows that the effect of salt on inhibiting root growth is mediated by JA [16]. Therefore, we wanted to know if *OsWRKY42* enhances salt stress tolerance by modulating expression of genes in the JA response pathway. The effect of salt on expression levels of JA biosynthesis and responsive genes was assessed by qPCR in one-week old Arabidopsis seedlings. The fold change was estimated by comparing with water treated controls for both wild type and 35S::OsWRKY42 transgenic lines. The expression levels of Arabidopsis JA biosynthesis genes like *Allene oxide cyclase3* (*AtAOC3*), *Lipoxygenase-2* (*AtLOX2*) and JA responsive genes like *Coronatine insensitive-1* (*AtCOI-1), Jasmonate ZIM motif 1 (AtJAZ1), AtJAZ10* and *Ethylene response factor* (*AtERF*) were tested in wild type and 35S::OsWRKY42 lines under salt stress. The expression of all the above JA biosynthesis and responsive genes was significantly (*p* < 0.05) downregulated in 35S::OsWRKY42 lines as compared to the wild type under salt stress (Fig. 5a). In the absence of any stress, the expression levels of the above genes in *OsWRKY42* expressing lines were similar to wild type (Additional file 5: Figure S4).

The levels of JA biosynthesis and response genes were also quantified in rosette stage leaves, 12 h post treatment with cellulase. Cellulase treatment induced expression of several JA biosynthesis and responsive genes (*AtAOC-3*, *AtCOI-1*, *AtJAZ1, AtJAZ10* and *AtERF*) (Fig. 5b). We observed that expression of the JA biosynthesis and responsive genes was significantly (*p* < 0.05) reduced in 35S::OsWRKY42 lines as compared to the wild type after cellulase treatment (Fig. 5b). Similarly, the expression of JA biosynthesis (*Allene oxide synthase*; *OsAOS1, Oxophytodienoate reductase-2; OsOPR2*) and response genes (*OsJAZ13, OsJAZ8, OsMYC*) was found to be significantly downregulated after transient overexpression of *OsWRKY42* in rice leaves (Fig. 5c). These genes had been previously shown to be upregulated at 12 h time point after ClsA treatment in rice leaves [9].

### *OsWRKY42* expressing Arabidopsis lines show tolerance to MeJA induced growth inhibition

**Fig. 6:**
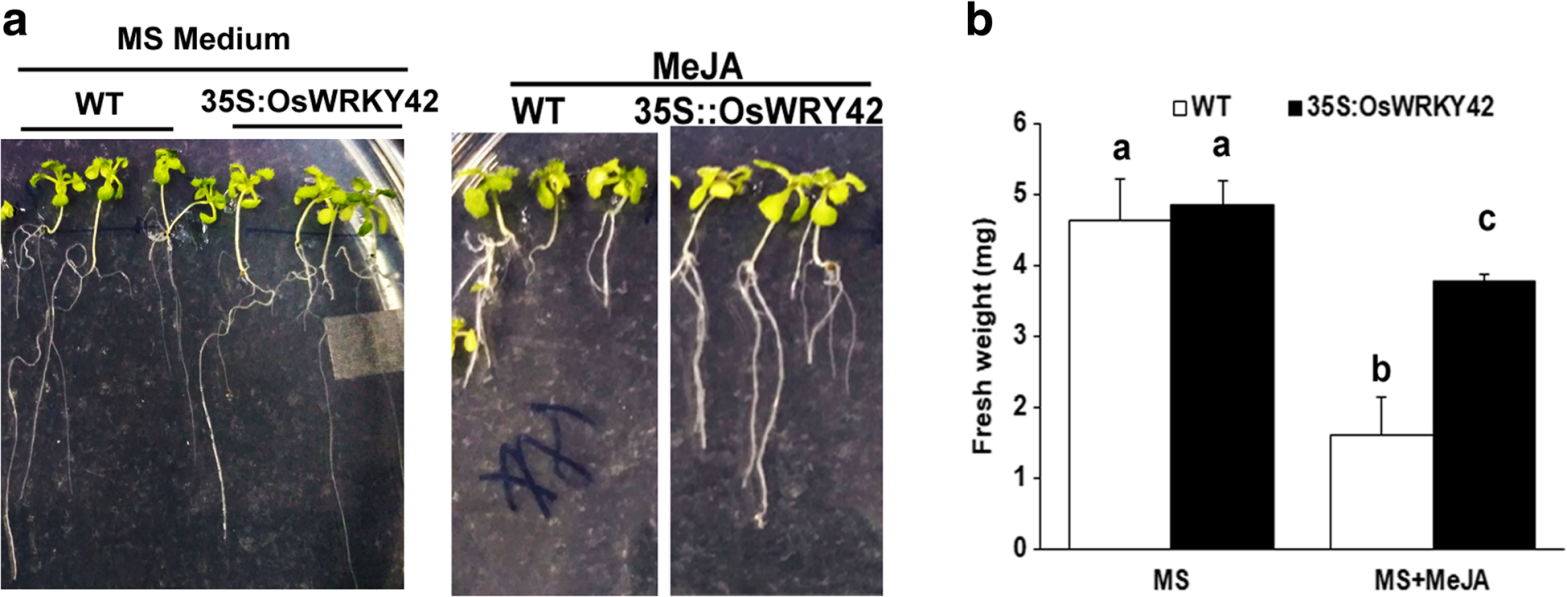
*OsWRKY42* expressing Arabidopsis transgenic lines are more tolerant to MeJA induced growth inhibition. The effect of MeJA on growth was studied in one-week-old Arabidopsis seedlings (wild type and 35S::OsWRKY42 lines). Seedlings (*n* = 10) were grown on MS agar medium with either MeJA (100 μM, Sigma-Aldrich) or DMSO (control). Growth was measured in the form of fresh weight on seventh day for each seedling (**a-b**). Data was analysed using one-way ANOVA followed by the Tukey-Karmer honestly significance difference test. The alphabets above the bar indicate significant differences with *p* value < 0.05. Presence of the same alphabet above the bar indicates that no significant difference was observed. The experiments were repeated three times in three independent 35S::OsWRKY42 transgenic lines

Four days old Arabidopsis seedlings (35S::OsWRKY42 and wild type) were grown on MS agar either in the presence or absence of 100 μM MeJA. Fresh weight of individual seedlings was measured after one week of MeJA treatment. In the presence of MeJA, there was approximately 60% reduction in fresh weight of wild type seedlings with respect to the seedlings grown on only MS (Fig. 6a). In contrast, the 35S::OsWRKY42 lines showed only 30% reduction in seedling weight compared to the control (Fig. 6b).

## Discussion

### *OsWRKY42* expression is induced following treatment with cell wall degrading enzymes in rice

We had earlier demonstrated that treatment of rice leaf tissues with CWDEs induces immune responses which enhances tolerance to subsequent infection with *Xoo* [6]. However, the rice functions involved in the regulation of this response are yet to be identified. In previously performed transcriptome analysis, *OsWRKY42* was the only transcription factor to be upregulated at early as well as at later time point after treatment with *Xoo* secreted LipA enzyme [10]. *OsWRKY42* expression is also induced on treatment with other CWDEs like xylanase and pectinase as well as after wounding. This suggests that damage to the plant cell wall induces expression of *OsWRKY42* in rice leaves*.* However*,* treatment with a bacterial PAMP such as Flg22 did not induce the expression of *OsWRKY42* (Fig. 1). Also, *OsWRKY42* was not upregulated in the transcriptome analysis done upon LPS treatment in rice leaves [17]. This indicates that *OsWRKY42* expression is induced following treatment with CWDEs but not after treatment with PAMPs such as Flg22 and LPS.

### *OsWRKY42* expression induces callose deposition but does not enhance tolerance to bacterial infection

Initially we hypothesized that *OsWRKY42* might be a positive regulator of CWDE induced immune responses. Therefore, callose deposition and bacterial infection assays were performed. Callose deposition is known to be associated with induction of immune responses [13, 18]. Treatment with PAMPs (Flg22, EF-Tu, chitin, LPS), DAMPs (Oligogalacturonides) and CWDEs has been shown to trigger callose deposition in plants [6, 17–21]. Overexpression of *OsWRKY42* in rice and Arabidopsis induced callose deposition but did not provide enhanced tolerance to bacterial infection suggesting that *OsWRKY42* might not be a positive regulator of immune responses. The composition of callose is a key factor in determining its role during pathogen invasion. It is possible that the composition of *OsWRKY42* induced callose deposits may not be potent enough to resist pathogen infection. *OsWRKY42* overexpression failed to induce expression of callose synthase genes (*AtGSL5,-6, − 7, − 12*) which are known to be associated with enhanced tolerance to microbial infection (Additional file 6: Figure S6) [18]. It is possible that enhanced callose deposition following *OsWRKY42* overexpression may result from enhanced expression of some other callose synthase or through post-transcriptional changes in expression of *AtGSL5,-6, − 7, − 12.*. In barley, the callose deposits that were not penetrated by powdery mildew fungi (*Blumeria graminis*) had significantly higher levels of arabinoxylan and cellulose along with the callose polymer as compared to the penetrated callose deposits [22, 23]. Modulation of the expression of genes involved in heteroxylan biosynthesis, lead to altered susceptibility to powdery mildew infection in barley [23]. Also, deposition of phytoalexin at the site of callose is associated with resistance to fungal infection [24]. Thus, we tested expression of genes involved in phytoalexin (*AtPAD3, AtCYB81F2, AtPEN2*) and heteroxylan biosynthesis genes [(*IRX9-L* (β-1,4 Xylosyl transferase), *GT61* (Glycosyl transferase family 61) *MUR3* (Xyloglucan galactosyl transferase)] in *OsWRKY42* expressing Arabidopsis transgenic lines*.* Expression of phytoalexin biosynthesis genes was not affected by expression of *OsWRKY42* but two genes involved in heteroxylan biosynthesis, namely *IRX9-L* and *MUR3,* were found to be downregulated by 1.5 to 2-fold upon *OsWRKY42* expression in Arabidopsis transgenic plants (Additional file 6: Figure S6). This supports the hypothesis that the *OsWRKY42* induced callose deposits may have a composition which does not promote enhanced tolerance to pathogen infection. Another possibility could be that the callose observed after overexpression of *OsWRKY42* may not be for preventing pathogen invasion but it could be a cell wall strengthening mechanism triggered after cell wall damage. Callose deposition is known to provide mechanical strength to the plasmalemma and the cell wall against different environmental abiotic stresses like drought, cold, heavy metal treatment and phosphorous deficiency [13, 25]. Therefore, it is possible that the callose deposition which is triggered by *OsWRKY42* expression may be for maintenance of cell wall integrity and not for enhancing tolerance against microbial infection.

### *OsWRKY42* expression enhances tolerance to salt stress in Arabidopsis

Publicly available microarray data indicates that expression of *OsWRKY42* is induced following abiotic stresses such as salinity. Our results indicate that treatment with NaCl enhances *OsWRKY42* expression in rice seedlings. Ectopic expression of *OsWRKY42* leads to enhanced tolerance to salt stress in Arabidopsis. Han and co-workers showed that *OsWRKY42* overexpressing rice transgenic lines exhibited elevated levels of ROS and early induction of senescence [26]. Our results indicate that *OsWRKY42* expressing Arabidopsis lines exhibit high basal levels of ROS but this had no apparent effect on plant growth. This suggests that *OsWRKY42* expressing Arabidopsis plants are able to overcome ROS induced oxidative damage through some as yet unidentified mechanisms. Furthermore, unlike in wild type plants the ROS levels did not increase in these plants after salt treatment. This may be because of the extensive anthocyanin production that is observed in *OsWRKY42* expressing lines under salt stress. During abiotic stress, antioxidant pigments like anthocyanins are produced to scavenge ROS [27, 28].

Treatment with either JA or salt is known to induce anthocyanin production in plants [27–29]. Salinity stress induces JA levels but there is no direct evidence to indicate that anthocyanin production during salinity stress is mediated by JA. In our experiments with wildtype Arabidopsis plants, salt treatment induces a JA response pathway but the salt stress does not induce anthocyanin production. *OsWRKY42* overexpressing lines have a high basal level of ROS. It is possible that the ROS levels in salt treated *OsWRKY42* overexpressing lines would have reached a much higher level if it were not scavenged by the enhanced production of anthocyanin that is observed under these conditions. Thus, in *OsWRKY42* expressing lines the plants produce anthocyanin to scavenge excess of ROS. There are many reports of JA independent mechanisms for anthocyanin production which include other hormones like cytokinin, ethylene, gibberellic acid and abscisic acid [30]. In the present study, the observed increase in anthocyanin production during salt stress may be independent of JA.

### *OsWRKY42* expression downregulates the levels of JA biosynthesis and responsive genes

Recent studies in rice and Arabidopsis indicates that salt stress mediated plant growth inhibition involves jasmonic acid signalling. Rice JA deficient mutants, *cpm2* and *hebiba*, exhibited enhanced tolerance to salt stress [31]. Overexpression of *OsCYP94,* a rice gene known to suppresses JA responses, increases tolerance to salt stress [32]. Also, in Arabidopsis, the mutants that are deficient either in JA biosynthesis or response (*aos, coi1, jaz3, myc2/3/4, jar1*) are more tolerant to salt stress mediated root growth inhibition [16]. These studies indicate that the JA response is induced during salt stress and that it is one of the causes for the suppression of plant growth under salt stress. Expression of *OsWRKY42* in Arabidopsis significantly downregulated salt induced expression of JA biosynthesis and responsive genes. Our previous studies show that in rice leaves, expression of genes involved in either JA biosynthesis or response is upregulated twelve hours after treatment with CWDEs (LipA/ClsA) in rice leaves [9, 10]. Similarly, in the present study we show that treatment of Arabidopsis leaves with cellulase induces expression of JA response genes. Expression of *OsWRKY42* in Arabidopsis significantly reduced cellulase induced expression of JA biosynthesis and response genes*.* We have also observed that transient expression of *OsWRKY42* in rice leaves suppressed the expression of JA response genes. Taken together, the above results suggest that *OsWRKY42* suppresses the expression of JA response pathway genes in rice and Arabidopsis. The observation that *OsWRKY42* expressing Arabidopsis plants exhibit enhanced tolerance to MeJA mediated growth suppression is consistent with this possibility.

SA and JA signalling pathways function in an antagonistic manner in Arabidopsis [33]. Ideally, downregulation of the JA response pathway by *OsWRKY42* should upregulate the SA response pathway and therefore provide enhanced tolerance to *Pst* infection. Our qPCR analysis shows that the levels of SA response genes (*AtPR2*, *AtSID2*) in *OsWRKY42* overexpressing lines remained same as that of the wildtype (Col-0) after *Pst* infection (Additional file 7: Figure S5). This indicated that the suppression of JA responsive genes by *OsWRKY42* is not enough to bring the SA-JA antagonistic effect. Thus, we didn’t observe any enhanced tolerance or susceptibility for *Pst* infection in Arabidopsis plants that are overexpressing *OsWRKY42*.

### *OsWRKY42* acts as a negative regulator of JA mediated stress responses in plants

**Fig. 7:**
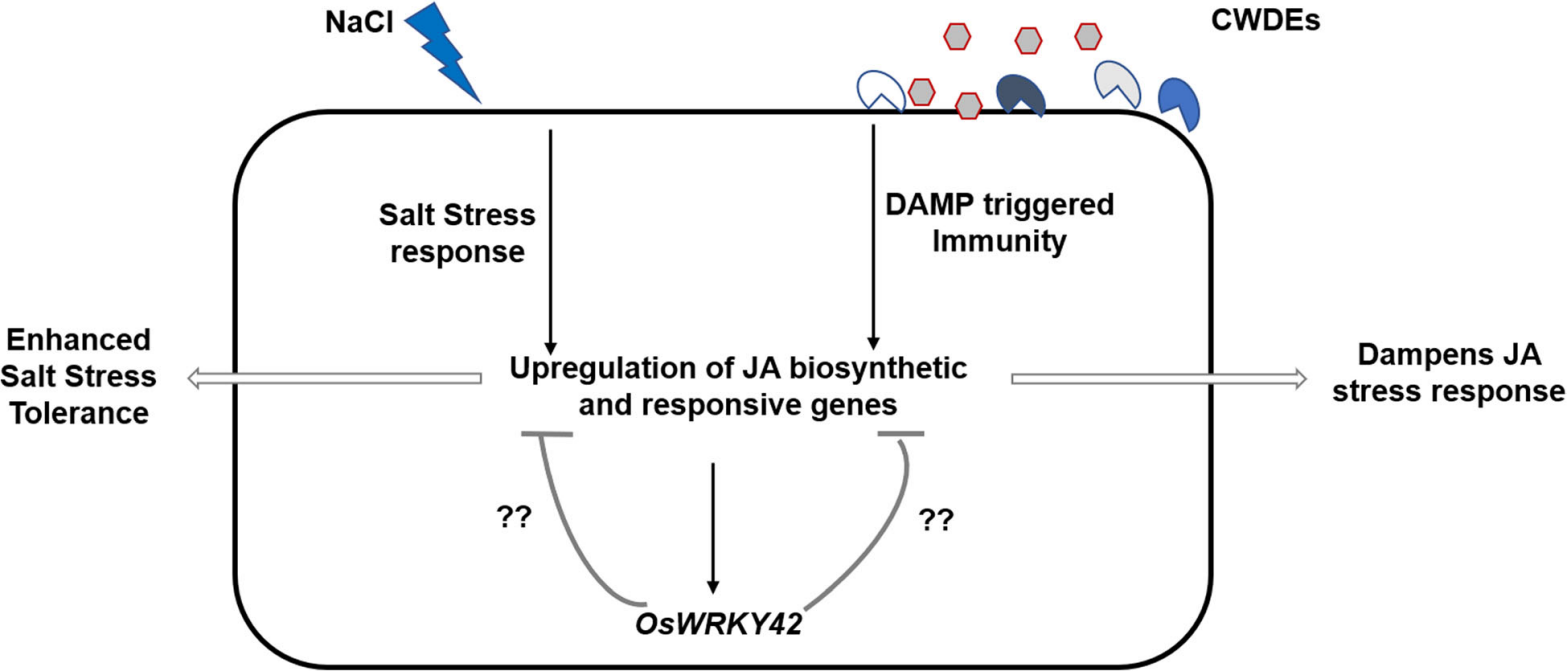
*OsWRKY42* is a negative regulator of JA mediated stress responses. Overexpression of *OsWRKY42* downregulates the expression of JA biosynthesis and responsive genes induced after treatment with either salt or any one of several different cell wall degrading enzymes (CWDEs). This results in enhanced tolerance to salt stress and in the dampening of JA mediated defense responses that are induced following cell wall damage

A continuously induced JA response can be deleterious to plants. For example, treatment with MeJA inhibits root growth in Arabidopsis [34]. Rice plants overexpressing *OsMYC2*, a JA response gene, exhibited a dwarf phenotype [35]. Overexpression of allene oxide cyclase in *Artemisia annua*, had a negative effect on plant growth [36]. Because of these deleterious effects on growth, JA induced responses need to be efficiently regulated. For tight regulation, JA itself induces the expression of its suppressors like JAZ and Arabidopsis bHLH class III transcription factor proteins [37, 38]. Also, treatment of rice seedlings with MeJA induces expression of *OsWRKY42* (Additional file 8: Figure S7). Here, we propose that *OsWRKY42* may act as a negative regulator of JA mediated responses that are induced following treatment with either CWDE or salt (Fig. 7.) and that CWDE induced *OsWRKY42* expression is intended to dampen JA responses.

The closest orthologues of *OsWRKY42* in Arabidopsis, *AtWRKY11* and *AtWRKY17*, with 42% and 40% amino acid sequence identity respectively, are also reported as negative regulators of *Pst* induced basal immune responses [39]. Chen and co-workers have quantitated levels of JA in *OsWRKY42* overexpressing rice transgenic lines and their results indicate that basal levels of JA are significantly lower in the transgenic lines as compared to wild type. It is not yet clear how *OsWRKY42* might suppress JA responses. It might do so by directly repressing expression of genes involved in JA biosynthesis/response or indirectly through repression of a positive activator of genes involved in JA biosynthesis/response.

## Conclusion

Enhanced expression of *OsWRKY42* following treatment with either CWDEs or salt may serve to dampen JA mediated stress responses.

## Methods

### Plant materials and growth conditions

Fourteen days old greenhouse grown seedlings of bacterial blight susceptible rice cultivar, Taichung Native-1 (TN-1), were used for callose deposition assays and for qRT-PCR analysis. Forty days old greenhouse-grown TN-1 plants were used for *Xoo* infection assays. *Arabidopsis thaliana* Columbia ecotype (Col-0) was used as wild type and also for the generation of transgenic plants. For all experiments, Arabidopsis seeds were surface sterilized with sterilization solution (70% ethanol containing 0.1% Tween-20) for fifteen minutes followed by five washes with sterile MilliQ purified water (MQ). Seeds were germinated on ½ MS (Murashige and Skoog’s) agar medium (pH 5.8). One-week-old Arabidopsis seedlings were placed into pots with soil (vermiculite: perlite: tissue culture mix in 1:1:2 ratio) and grown in a plant growth chamber (Percival) set at 22 °C with a 12 h/12 h light/day cycle.

### Bacterial strains and growth conditions

*Agrobacterium tumefaciens* strain LBA4404 was used for rice transient transformation assays and for generation of Arabidopsis transgenic plants. The LB4404 strain derivatives carrying different plant expression vectors (Additional file 9: Table S3) were grown overnight at 28 °C in Luria Bertani (LB; HiMedia) broth. *Xoo* strain BXO43 (our laboratory wild type) was grown and maintained on PSA (peptone 10 g L^− 1^, sucrose 10 g L^− 1^, agar 12 g L^− 1^) medium with rifampicin (25 mgL-1). *Pseudomonas syringae* pv. *tomato* (*Pst*) DC3000 was maintained on King’s basal agar medium (HiMedia) and for plant inoculations the culture was grown overnight at 28 °C in LB broth with rifampicin (25 mgL-1).

### Generation of plant expression plasmids (pMDC7::OsWRKY42 and pH 7FWG2::OsWRKY42) using gateway cloning system

The *OsWRKY42* gene (LOC_Os02g26430) encodes a CDS of length 762 bp. The cDNA was prepared using Superscript III reverse transcriptase (Invitrogen) from total RNA isolated from LipA treated TN-1 rice leaves (2 h post-treatment) as described previously [10]. The *OsWRKY42* gene with 2 x FLAG tag was cloned into the inducible plant expression vector, pMDC7, by Gateway cloning as per the manufacturer’s instructions (Invitrogen). In pMDC7, gene expression is under the control of the 17-β estradiol inducible XVE promoter. For generation of C-terminally GFP tagged *OsWRKY42* clones, the gene was cloned without stop codon into pH 7FWG2 plant expression vector. The gene was expressed constitutively under CaMV35S promoter and with GFP tag at the C-terminal region of the protein. These plant expression vectors containing *OsWRKY42* were transformed into *A. tumefeciens* LBA4404 strain by electroporation and selected on medium containing appropriate antibiotics (Additional file 9: Table S3). The LBA4404/pMDC7::OsWRKY42 clones were confirmed by colony PCR and subsequent sequencing of the PCR amplicons using vector specific primers (Additional file 10: Table S2).

### Quantitative real time PCR (qRT-PCR) analysis

Total RNA from either Arabidopsis or rice leaves was isolated using TRIzol Reagent (Thermo Fisher Scientific). The quality of RNA was checked on agarose gels followed by DNaseI (NEB) treatment as per the manufacturer’s instructions. cDNA was synthesized with 1 μg of total RNA with oligo (dT)-primer using RNA to cDNA EcoDry premix kit (Clontech, Takara). One microliter of the 1:10-fold diluted cDNA was subjected to qRT-PCR using Power SYBR Green/ROX Master Mix (Thermo Fisher Scientific) on the 7900 HT sequence detection system (Applied Biosystems, USA). *OsGAPDH* and *AtUBQ5* were used as internal controls for rice and Arabidopsis, respectively. The primers used for qPCR (Additional file 10: Table S2) were designed using Quantprime [40]. The relative expression of various genes between test and control samples was calculated using the 2 ^-ΔΔCt^ method [41].

### Biotic and abiotic stress treatments in Rice

Leaves of fourteen days old TN-1 plants were infiltrated with one of the following: xylanase (2 U/ml, Sigma), pectinase (2 U/ml, Sigma), Flg22 (100 μM, Genescript) and water. For assaying response to wounding, leaves of fourteen days old TN-1 rice seedlings were pin pricked 8–10 times and sprayed with water [7]. Leaves were harvested 12 h post-treatment and processed for qRT-PCR as mentioned previously. For abiotic stress treatment, one-week-old TN-1 rice seedlings were dipped in each of the following: 150 mM NaCl solution (salinity stress), a 20% (*w*/*v*) PEG-6000 (drought stress) and water (mock). The treated seedlings were incubated in a greenhouse. Leaves were harvested after 12 h of treatment and processed for qRT-PCR as mentioned previously. In order to study the effect of *OsWRKY42* on expression of JA response genes, transient overexpression of *OsWRKY42* was carried out. For this, leaves of fourteen days old TN-1 rice seedlings were infiltrated either with LBA4404 or LBA4404/pH7FWG2::OsWRKY42*.* Leaves were harvested 12 h post treatment and processed for qRT-PCR analysis.

### Callose deposition

For callose deposition assays in rice, *OsWRKY42* was transiently overexpressed in leaves of fourteen days old TN-1 seedlings. LB4404/pMDC7::*OsWRKY42* was grown overnight at 28 °C in LB broth. The culture was washed with sterilized water and resuspended (OD_600_ = 0.8) in induction solution [10 mM MES (Sigma), pH 5.6 with 200 μM acetosyringone (HiMedia)]. Rice leaves were infiltrated with the culture either with or without 17-β estradiol (20 μM, Sigma) using a 1 ml needleless syringe. Leaves were harvested 16 h after infiltration and stained for callose. Harvested leaves were fixed in ethanol:acetic acid (3:1) solution for 4 h and the solution was changed regularly until all of the chlorophyll pigment was removed. The leaves were rehydrated in 70% ethanol followed by 50% ethanol, each for 2 h and then in water for 2 h. The leaves were stained overnight in 0.01% (w/v) aniline blue solution in potassium phosphate buffer (150 mM; pH 9.5) (Millet et al., 2010). Callose deposits were observed and imaged with an epifluorescence microscope (ECLIPSE Ni-E, Nikon) using blue filter (excitation wavelength 365 nm) under 10× magnification. The number of callose deposits per field of view from at least six leaves with eight different fields viewed per leaf were counted in each experiment. Three independent experiments were performed.

Callose deposition was also assayed in T_2_ generation of transgenic Arabidopsis lines (XVE::OsWRKY42) overexpressing *OsWRKY42* under a 17-β-estradiol inducible system. Rosette stage leaves of three weeks old plants were infiltrated either with the inducer (20 μM 17-β-estradiol) or water (mock) using a 1 ml needleless syringe. Sixteen hours post infiltration, the leaves were harvested and processed as mentioned above.

### Western-blotting

Leaves of fourteen days old TN-1 rice seedlings were infiltrated with saturated Agrobacterial cultures of LBA4404/ pMDC7::OsWRKY42 with or without estradiol. After 16–18 h, the infiltrated region was cut and ground in liquid nitrogen followed by homogenization in lysis buffer (50 mM Tris-HCl [pH 7.5], NaCl [150 mM], Mannitol [250 mM], EDTA [5 mM], 10% Glycerol, 1 mM DTT 1% TritonX100, 1 mM PMSF, 1 mM NaF) with plant protease inhibitor cocktail (Sigma-Aldrich) [42]. Total protein supernatants were isolated after centrifugation at 15000 g for 15 min at 4 °C to remove cellular debris. Equal amounts of isolated protein supernatants were separated using 10% sodium dodecyl sulfate polyacrylamide gel electrophoresis (SDS-PAGE). The OsWRKY42 protein was detected by Western blot analysis using anti-FLAG (AbCAM) antibodies. HRP conjugated anti-rabbit secondary antibody (AbCAM) was probed and the protein band was viewed using Luminata Forte HRP substrate (Millipore). The signal was captured under Vilber Lourmat chemiluminescence imaging system with Chemi-capt 5000 software (version 12.8; Vilber Lourmat).

### *Xoo* infection of rice

For transient overexpression of *OsWRKY42* in TN-1 rice leaves, Agrobacterial strain LBA4404/pMDC7::OsWRKY42 was grown overnight in LB broth at 28 °C. The culture was washed once with sterile water and resuspended (OD_600_ = 0.8) in induction solution (as mentioned above). The midveins of the leaves of 40 days old rice plants were injected with the bacterial suspension, using a 1 ml syringe with or without estradiol. Twelve hours later, the midveins of the leaves were infected with wild type *Xoo* (BXO43 strain) by pricking with a needle touched to a fresh bacterial colony grown on PSA (peptone 10 g L^− 1^, sucrose 10 g L^− 1^, agar 12 g L^− 1^). Lesion lengths were measured on the tenth day post infection.

### Generation of transgenic Arabidopsis plants

Arabidopsis ecotype Col-0 (wild type) was transformed with *Agrobacterium* strain LBA4404 carrying either pMDC7::OsWRKY42 or pH 7FWG2::OsWRKY42 using the floral dip method [43]. Transgenic seedlings were selected on MS agar medium containing hygromycin (25 μg/ml). The transgenic nature of the T_1_ plants was further confirmed by direct PCR (Terra PCR direct polymerase kit, Clontech using leaf tissue and sequencing of the amplified product. The T_2_ progeny of three independent transgenic lines were used for functional studies. Assays for callose deposition and bacterial infection were performed using inducible pMDC7::OsWRKY42 transgenic lines (XVE::OsWRKY42) that expressed high levels of *OsWRKY42*. Transgenic lines that constitutively expressed *OsWRKY42* were generated using pH 7FWG2::OsWRKY42 and used for salt stress response studies.

### *P. syringae* infection in Arabidopsis plants

Twelve hr. prior to inoculation, leaves of 4-week-old plants were pre-infiltrated with either estradiol (20 μM) or water using a needleless syringe. For *Pst* infection, bacteria were grown overnight at 28 °C in LB broth with rifampicin (25 mgL-1). The culture was washed with sterile water and resuspended (OD600 = 0.01) in 10 mM MgCl_2_ solution. The above suspension was infiltrated in pre-treated (estradiol or water) leaves using a needleless 1 ml syringe. Disease progression was monitored by measuring bacterial growth in leaves as mentioned previously [44]. Briefly, infected leaves that were harvested on different days post infection (0–2-4 dpi) were homogenized in 10 mM MgCl_2_ and diluted serially for assaying total bacterial counts. Each data point consisted of five replicates.

### Salt treatment in Arabidopsis seedlings

Responses to salt stress were studied in either wild type or T_2_ generation of transgenic Arabidopsis lines (35S::OsWRKY42) constitutively expressing *OsWRKY42.* The transgenic seedlings (*n* = 20) were selected on hygromycin containing MS agar. One-week-old seedlings (35S::OsWRKY42 or wild type) were transferred to MS agar medium with or without NaCl (150 mM, Merck). The number of root branches and the total fresh weight (mg) of seedlings was measured on fifteenth day post-treatment. The effect of salt on growth of *OsWRKY42* transgenic lines and wild type (Col-0) plants was compared. The experiments were repeated thrice in three independent transgenic lines.

Expression of JA biosynthesis and response genes was analysed after salt treatment in constitutively expressing 35S::OsWRKY42 transgenic lines and wild type using quantitative real-time PCR (qRT-PCR). For this, one-week old seedlings (n = 20) were placed on sterile filter paper soaked in MS solution either in the presence or absence of NaCl (150 mM). Twelve hours post treatment the seedlings were processed for qRT-PCR analysis.

### Anthocyanin estimation

Three weeks old Arabidopsis plants (wild type and 35S::OsWRKY42 constitutive lines) were treated either with 150 mM NaCl or with water for fifteen days. On the fifteenth day, leaves were weighed and crushed in liquid nitrogen. Anthocyanin was extracted in acidic methanol (1% HCl in methanol) and absorbance of the extract was measured at 530 nm and 657 nm using an Eppendorf Biospectrophotometer. The anthocyanin content per milligram of leaf tissue was calculated [31].

### ROS estimation by NBT assay in Arabidopsis leaves

Leaf discs of three weeks old Arabidopsis plants that are either wild type or constitutively expressing *OsWRKY42* (35S::OsWRKY42) were placed on sterile filter paper soaked in MS solution either with or without NaCl (150 mM). Twelve hours post treatment, the leaves were immersed in 2 ml of NBT solution [0.05% NBT (*w*/*v*) and 10 mM NaN_3_ dissolved in 10 mM potassium phosphate buffer pH 7.8] and incubated at room temperature for one hour [45]. After incubation, the solution was boiled at 85 °C for 15 min and cooled immediately to stop the reaction. The absorbance was measured at 580 nm using an Eppendorf Biospectrophotometer. The O_2_^−^ content per milligram of tissue was calculated.

### Cellulase treatment in Arabidopsis leaves

Leaves of 3-weeks-old plants (wild type and 35S::OsWRKY42 constitutive lines) were infiltrated with cellulase (2 U, Sigma) or water using a needleless syringe. Leaves were harvested 12 h post treatment and processed for qRT-PCR as mentioned previously.

### Methyl jasmonic acid (MeJA) treatment of Arabidopsis seedlings

Growth response to MeJA treatment was studied in T_2_ generation of Arabidopsis lines that were either transgenic (35S::OsWRKY42) or wild type. One-week-old seedlings (*n* = 10) were grown on MS agar medium containing either MeJA (100 μM, Sigma-Aldrich) or DMSO (control). The growth of individual seedlings was measured (fresh weight) on the seventh day post-treatment. The experiments were repeated thrice in three independent transgenic lines.

### Statistics

Unless mentioned, all experiments were performed at least in three biological replicates. All experiments in Arabidopsis were reproduced in three independent transgenic lines. Statistical analysis was performed using the Student’s *t*-test in Microsoft Excel software. One-way ANOVA followed by the Tukey-Karmer honestly significance difference test was used for analysing data obtained after salt and MeJA treatment in Arabidopsis seedlings.

## Additional files

Additional file 1: **Figure S1.** Transient expression of *OsWRKY42* in rice leaves was confirmed by Western blotting. The leaves (*n* = 10) of fourteen days old rice seedlings were syringe infiltrated with Agrobacterium strain LBA4404/pMDC7::OsWRKY42 along with 20 μM 17-β Estradiol (Est)/ Water. Leaves were collected after 16 h and crushed in protein extraction buffer and processed for Western blotting. The OsWRKY42 protein was detected using anti-FLAG antibody (approximate size is 29 kDa). (TIF 470 kb)

Additional file 2: **Figure S2.** Estradiol inducible expression of *OsWRKY42* in XVE::OsWRKY42 transgenic Arabidopsis plants. Leaves of three weeks old plants were infiltrated either with inducer (20 μM 17-β-estradiol) or water using a 1 ml needleless syringe. Sixteen hours post infiltration, leaves were harvested and processed for qPCR analysis. The graph represents relative fold change (2^-∆∆Ct^) using expression values of Est treated over water treated samples. *AtUBQ5* was used as an internal control for qPCR analysis. The error bar represents standard deviation. (TIF 3271 kb)

Additional file 3: **Table S1.** *OsWRKY42* expression is induced under various biotic as well as abiotic stresses. (DOCX 17 kb)

Additional file 4: **Figure S3.** Ectopic expression of OsWRKY42-GFP protein in the constitutive 35S::OsWRKY42 was confirmed by microscopy. Expression of OsWRKY42-GFP protein in the constitutive 35S::OsWRKY42 Arabidopsis transgenic lines was visualised under an epifluorescence microscope. One-week old 35S::OsWRKY42 seedlings were directly placed on mounting medium and observed under GFP filter and DIC using an epifluorescence microscope. The image shown here is the apical region of a root tip showing expression of GFP-tagged OsWRKY42. The scale bar represents 20 μm. (TIF 1956 kb)

Additional file 5: **Figure S4.** Constitutive expression of *OsWRKY42* had no effect on levels of JA biosynthesis and response genes in 35S::OsWRKY42 Arabidopsis transgenic lines that are not subjected to stress. Leaves from three weeks old Arabidopsis plants that are either wild type or transgenic for 35S::OsWRKY42 were harvested and processed for qPCR analysis. *AtUBQ5* was used as an internal control for qPCR analysis. The graph represents relative fold change (2^-∆∆Ct^) using expression values of 35S::OsWRKY42 over wild type plants. The average value from three biological samples is plotted in the graph. The error bar represents standard deviation. The experiments were repeated in three independent 35S::OsWRKY42 transgenic lines. (TIF 993 kb)

Additional file 6: **Figure S6.** Ectopic expression of *OsWRKY42* in Arabidopsis alters expression of genes involved in heteroxylan biosynthesis but does not affect expression of different callose synthase genes. Leaves of three weeks old plants were infiltrated either with inducer (20 μM 17-β-estradiol) or water using a 1 ml needleless syringe. Twelve hours post infiltration, leaves were harvested and processed for qPCR analysis. The graph represents relative fold change (2^-∆∆Ct^) using expression values of Est treated over water treated samples. *AtUBQ5* was used as an internal control for qPCR analysis. The error bar represents standard deviation. All of the above experiments were repeated in two independent transgenic lines. (TIF 2938 kb)

Additional file 7: **Figure S5.** Ectopic expression of *OsWRKY42*does not alter expression of SA responsive genes in *Pst* infected Arabidopsis leaves. Leaves of 35S::OsWRKY42 transgenic and wildtype (Col-0) Arabidopsis plants were infiltrated with cells of a *Pst* culture (OD = 0.01). Samples (3 leaves per plant) were collected, twelve hours post infection and processed for qRT-PCR using primers that are specific for SA responsive genes *AtPR2* and *AtSID2*. The graph represents relative fold change (2^-∆∆Ct^) using expression values of *Pst* infected over uninfected samples. *AtUBQ5* was used as the endogenous control. The average from three biological replicates is plotted on the graph. The error bar represents standard deviation. (TIF 1135 kb)

Additional file 8: **Figure S7.** Expression of OsWRKY42 is induced upon MeJA treatment. Leaves of fourteen days old rice seedlings were sprayed with either water or MeJA(100 μM). Four hours post treatment, leaves were harvested and processed for qRT-PCR. The relative fold change was calculated over water treated control. *OsGAPDH* was used as the endogenous control. Graph represents the mean from three biological replicates and error bar represents standard deviation. (TIF 715 kb)

Additional file 9: **Table S3.** List of bacterial strains and plasmids. (DOCX 17 kb)

Additional file 10: **Table S2.** List of primers. (DOCX 24 kb)

## Abbreviations

ClsA: CellulaseA
CWDE: cell wall degrading enzyme
DAMP: Damage associated molecular patterns
DTI: DAMP triggered immunity
Est: 17 β-Estradiol
GFP: Green fluorescent protein
JA: Jasmonic acid
LipA: Lipase/esterase A
MS: Murashige and Skoog’s
*Pst*: *Pseudomonas syringae* pv. *tomato* DC3000
ROS: Reactive oxygen species
TN-1: Taichung Native-1
*Xoo*: *Xanthomonas oryzae* pv. *oryzae*
