# Supplementary figures and images for: Overexpression of a cell wall damage induced transcription factor, OsWRKY42, leads to enhanced callose deposition and tolerance to salt stress but does not enhance tolerance to bacterial infection
Source: BMC Plant Biol. 2018 Sep 3;18:177. doi: 10.1186/s12870-018-1391-5 (PMC6122458; doi:10.1186/s12870-018-1391-5)

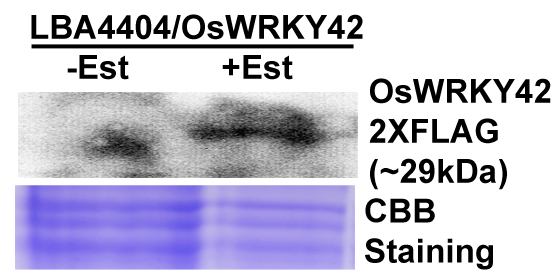

Supplement: Supplementary file 1 — Figure S1. Transient expression of OsWRKY42 in rice leaves was confirmed by Western blotting. The leaves (n = 10) of fourteen days old rice seedlings were syringe infiltrated with Agrobacterium strain LBA4404/pMDC7::OsWRKY42 along with 20 μM 17-β Estradiol (Est)/ Water. Leaves were collected after 16 h and crushed in protein extraction buffer and processed for Western blotting. The OsWRKY42 protein was detected using anti-FLAG antibody (approximate size is 29 kDa). (TIF 470 kb) [file 12870_2018_1391_MOESM1_ESM.tif]

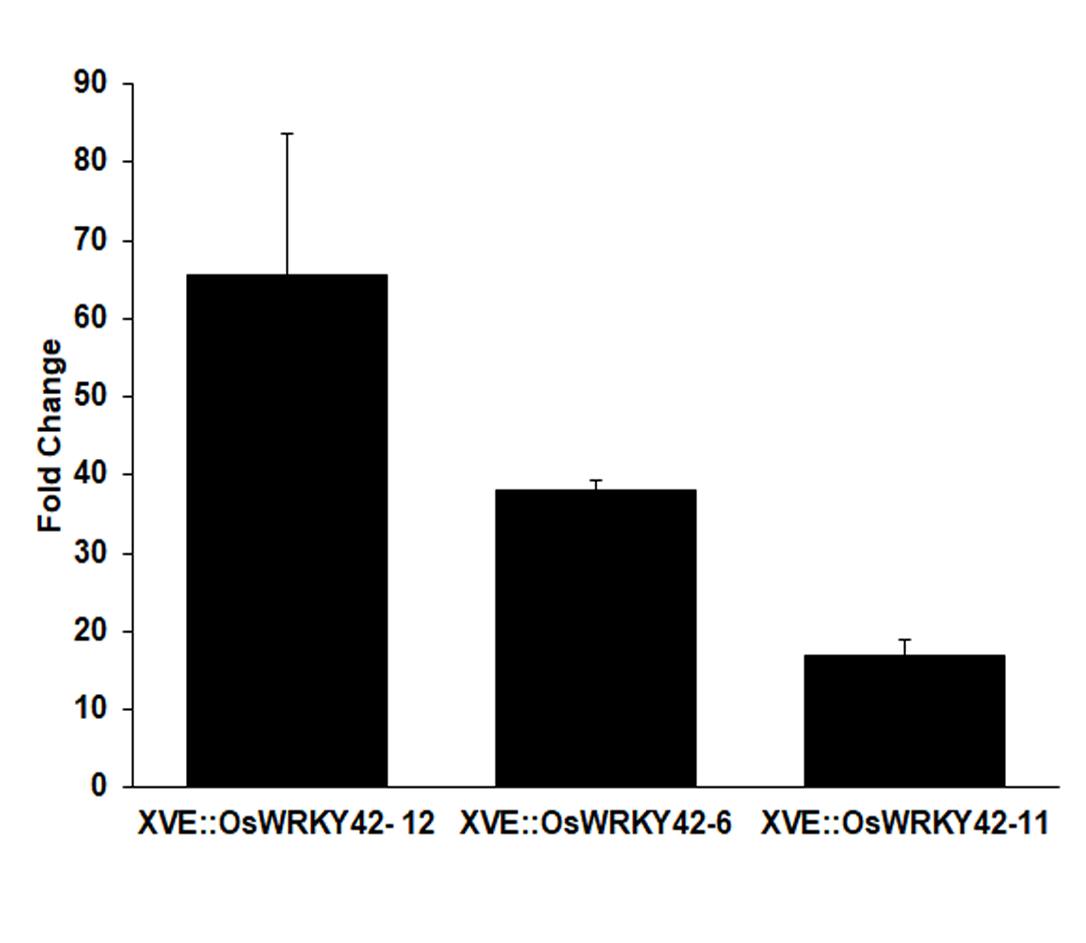

Supplement: Supplementary file 2 — Figure S2. Estradiol inducible expression of OsWRKY42 in XVE::OsWRKY42 transgenic Arabidopsis plants. Leaves of three weeks old plants were infiltrated either with inducer (20 μM 17-β-estradiol) or water using a 1 ml needleless syringe. Sixteen hours post infiltration, leaves were harvested and processed for qPCR analysis. The graph represents relative fold change (2-∆∆Ct) using expression values of Est treated over water treated samples. AtUBQ5 was used as an internal control for qPCR analysis. The error bar represents standard deviation. (TIF 3271 kb) [file 12870_2018_1391_MOESM2_ESM.tif]

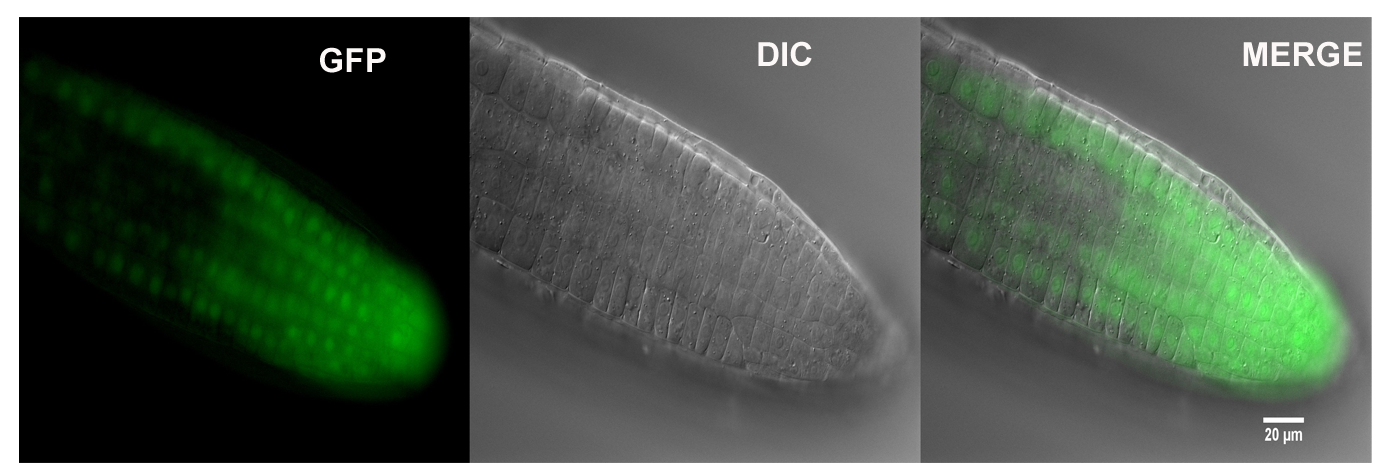

Supplement: Supplementary file 3 — Table S1. OsWRKY42 expression is induced under various biotic as well as abiotic stresses. (DOCX 17 kb) [file 12870_2018_1391_MOESM3_ESM.docx]

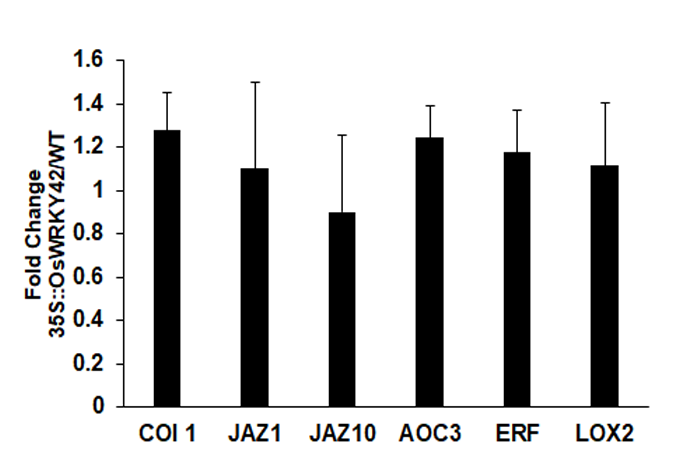

Supplement: Supplementary file 4 — Figure S3. Ectopic expression of OsWRKY42-GFP protein in the constitutive 35S::OsWRKY42 was confirmed by microscopy. Expression of OsWRKY42-GFP protein in the constitutive 35S::OsWRKY42 Arabidopsis transgenic lines was visualised under an epifluorescence microscope. One-week old 35S::OsWRKY42 seedlings were directly placed on mounting medium and observed under GFP filter and DIC using an epifluorescence microscope. The image shown here is the apical region of a root tip showing expression of GFP-tagged OsWRKY42. The scale bar represents 20 μm. (TIF 1956 kb) [file 12870_2018_1391_MOESM4_ESM.tif]

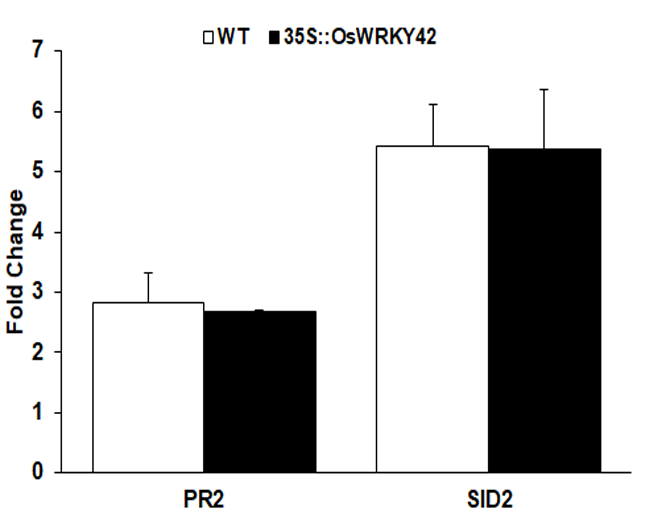

Supplement: Supplementary file 5 — Figure S4. Constitutive expression of OsWRKY42 had no effect on levels of JA biosynthesis and response genes in 35S::OsWRKY42 Arabidopsis transgenic lines that are not subjected to stress. Leaves from three weeks old Arabidopsis plants that are either wild type or transgenic for 35S::OsWRKY42 were harvested and processed for qPCR analysis. AtUBQ5 was used as an internal control for qPCR analysis. The graph represents relative fold change (2-∆∆Ct) using expression values of 35S::OsWRKY42 over wild type plants. The average value from three biological samples is plotted in the graph. The error bar represents standard deviation. The experiments were repeated in three independent 35S::OsWRKY42 transgenic lines. (TIF 993 kb) [file 12870_2018_1391_MOESM5_ESM.tif]

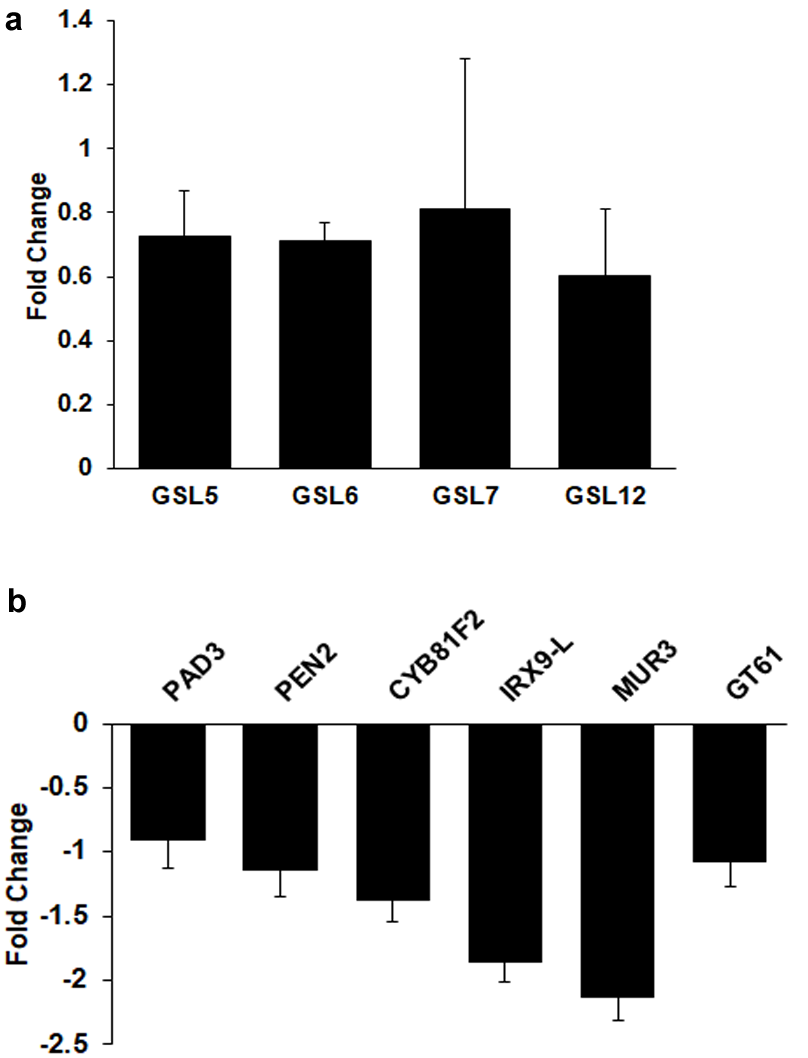

Supplement: Supplementary file 6 — Figure S6. Ectopic expression of OsWRKY42 in Arabidopsis alters expression of genes involved in heteroxylan biosynthesis but does not affect expression of different callose synthase genes. Leaves of three weeks old plants were infiltrated either with inducer (20 μM 17-β-estradiol) or water using a 1 ml needleless syringe. Twelve hours post infiltration, leaves were harvested and processed for qPCR analysis. The graph represents relative fold change (2-∆∆Ct) using expression values of Est treated over water treated samples. AtUBQ5 was used as an internal control for qPCR analysis. The error bar represents standard deviation. All of the above experiments were repeated in two independent transgenic lines. (TIF 2938 kb) [file 12870_2018_1391_MOESM6_ESM.tif]

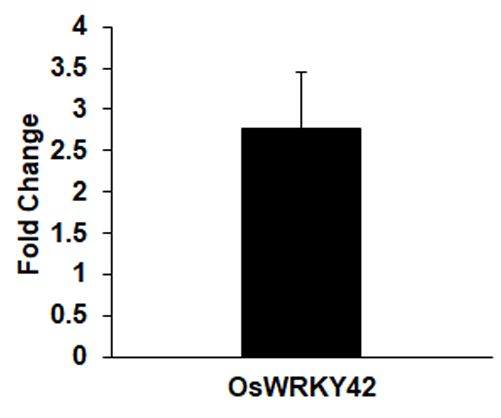

Supplement: Supplementary file 7 — Figure S5. Ectopic expression of OsWRKY42does not alter expression of SA responsive genes in Pst infected Arabidopsis leaves. Leaves of 35S::OsWRKY42 transgenic and wildtype (Col-0) Arabidopsis plants were infiltrated with cells of a Pst culture (OD = 0.01). Samples (3 leaves per plant) were collected, twelve hours post infection and processed for qRT-PCR using primers that are specific for SA responsive genes AtPR2 and AtSID2. The graph represents relative fold change (2-∆∆Ct) using expression values of Pst infected over uninfected samples. AtUBQ5 was used as the endogenous control. The average from three biological replicates is plotted on the graph. The error bar represents standard deviation. (TIF 1135 kb) [file 12870_2018_1391_MOESM7_ESM.tif]
