# Supplementary material for: Overexpression of a cell wall damage induced transcription factor, OsWRKY42, leads to enhanced callose deposition and tolerance to salt stress but does not enhance tolerance to bacterial infection
Source: BMC Plant Biol. 2018 Sep 3;18:177. doi: 10.1186/s12870-018-1391-5 (PMC6122458; doi:10.1186/s12870-018-1391-5)
Supplement: Supplementary file 8 — Figure S7. Expression of OsWRKY42 is induced upon MeJA treatment. Leaves of fourteen days old rice seedlings were sprayed with either water or MeJA(100 μM). Four hours post treatment, leaves were harvested and processed for qRT-PCR. The relative fold change was calculated over water treated control. OsGAPDH was used as the endogenous control. Graph represents the mean from three biological replicates and error bar represents standard deviation. (TIF 715 kb) [file 12870_2018_1391_MOESM8_ESM.tif]

**Table S1. *OsWRKY42* expression is induced under various biotic as well as abiotic stresses.**

| **Treatments** | **Time point^a^** | **Fold Change^b^** | **Rice variety** | **GEO accession number** |
| --- | --- | --- | --- | --- |
| **Biotic Stress** |  |  |  |  |
| *Magnaporthe grisea* FR13 | 3dpi | 6.68 | Nipponbare | GSE7256 |
| *Magnaporthe oryzae* guy11 | 2 dpi | 23 | Nipponbare | GSE18361 |
| *Xanthomonas oryzae* pv*. oryzae* PXO99a | 24 hr | 1.9 | IR24 | GSE36272 |
| *Xanthomonas oryzae* pv*. oryzae* PXO341 | 24 hr | 1.58 | IR24 | GSE36093 |
| *Xanthomonas oryzae* pv*. oryzicola* | 24 hr | 1.52 | IR24 | GSE36272 |
| Rice stripe virus (RSV) | 2 dpi | 3.45 | WunYun3 | GSE11025 |
| **CWDEs** |  |  |  |  |
| ClsA (cellulaseA of *Xoo* BXO43 strain) | 12 hr | 1.9 | TN-1 | GSE8216 |
| LipA (lipaseA of *Xoo* BXO43 strain) | 2 hr | 1.47 | TN-1 | GSE53940 |
| LipA (lipaseA of *Xoo* BXO43 strain) | 12 hr | 3.17 | TN-1 | GSE49242 |
| **Abiotic Stress** |  |  |  |  |
| Salinity stress | 3 hr | 2.08 | IR64 | GSE6901 |
| Drought stress | 3 hr | 5.7 | IR64 | GSE6901 |

Analysis of publicly available rice microarray data was performed for *OsWRKY42* using Genevestigator with Affymetrix probe set ID (Os.56922.1.S1_at).

^a^dpi: days post infection, hpi: hours post infection,

^b^ The fold change values are statistically significant (p ≤ 0.05).
