# Supplementary material for: Overexpression of a cell wall damage induced transcription factor, OsWRKY42, leads to enhanced callose deposition and tolerance to salt stress but does not enhance tolerance to bacterial infection
Source: BMC Plant Biol. 2018 Sep 3;18:177. doi: 10.1186/s12870-018-1391-5 (PMC6122458; doi:10.1186/s12870-018-1391-5)
Supplement: Supplementary file 9 — Table S3. List of bacterial strains and plasmids. (DOCX 17 kb) [file 12870_2018_1391_MOESM9_ESM.docx]

**Table S2: List of primers**

| Primers used for cloning and sequencing | |  |
| --- | --- | --- |
| Name (Use) | Primer sequence (5’ to 3’) |  |
| OsWRKY42FLF | CACCATGGCGGATCCGTTCCCGGC | This work |
| OsWRKY42FLR | GAGCAATCTTCCAGTAGTTTGGTCAG | This work |
| OsWRKY42FLAGR | CTAGACTACAAAGACGATGACGACAAGGAGCAATCTTC | This work |
| M13 F (Sequencing) | GTAAAACGACGGCCAGT |  |
| M13 R (Sequencing) | GGAAACAGCTATGACCATG |  |
| pMDC7 F | CAGCAGTCGAGGTAAGAT |  |
| pMDC7 R | GGTGTGTGGGCAATGAAA |  |
| T7-F (Sequencing) | TAATACGACTCACTATAGGG |  |
| T7-R (Sequencing) | GCTAGTTATTGCTCAGCGG |  |
| **qPCR primers used for rice genes** | |  |
| Genes | Primer sequence (5’ to 3’) |  |
| OsAOS-3F | CGTGTACTGGTCGAATGGGC | Jha et al., 2010 |
| OsAOS-3R | GTGAAGGTGTCGTACCGGAG | Jha et al., 2010 |
| OsJAZ8 F | GAAGGCTCAACAGCTGACCATAT | This work |
| OsJAZ8 R | TTGGTGGACGGGAAGTTCTC | This work |
| OsJAZ13 F | CGTGAGGATGCTTATTATGCTTG | This work |
| OsJAZ13 R | CCAATGAAATTATATGATCCCTAGC | This work |
| OsOPR2F | TACACGGACTACCCGTTTCT | Jha et al., 2010 |
| OsOPR2R | CGTAAGGTCGACCACAAACT | Jha et al., 2010 |
| OsMYCF | ACCAGCACGCTCACGGAGAAT | Jha et al., 2010 |
| OsMYCR | TGGACGCGAAGTCGGAGAAGT | Jha et al., 2010 |
| OsAOCF | CGTGTACTGGTCGAATGGGC | This work |
| OsAOCR | GTGAAGGTGTCGTACCGGAG | This work |
| OsWRKY42RTF | [ACGACTATTCGTGGCGCAAG](about:blank) | This work |
| OsWRKY42RTR | [CTTGTAGTACCCGCGTGGATAAG](about:blank) | This work |
| OsGAPDHF | GAGTATGATGAGTCGGGTCCAG | Jha et al., 2010 |
| OsGAPDHR | ACACCAACAATCCCAAACAGAG | Jha et al., 2010 |
| **qPCR primers used for Arabidopsis genes** | |  |
| Name | Primer sequence (5’ to 3’) |  |
| AtUBQ5 F | AAGAAGACTTACACCAAGCCGAAG | This work |
| AtUBQ5 R | ACAGCGAGCTTAACCTTCTTATGC | This work |
| AtAOS F | GGTGGCGAGGTTGTTTGTGATTG | This work |
| AtAOS R | TTCCTAACGGCGACGTACCAAC | This work |
| AtAOC3F | ACGTGTATGAGCTCAACGAAGGAG | This work |
| AtAOC3R | TCTTGCCGAGTTTAAGAACTGCTG | This work |
| AtCOI-1F | TGCACTTCCGACGGATGATTGTC | This work |
| AtCOI-1R | TCCTGCAGTGTGTAACGATGCTC | This work |
| AtERF1F | TCCCTTCAACGAGAACGACTCAG | This work |
| AtERF1R | AGGTTTGTTGCGTGGACTGCTC | This work |
| AtLOX2F | TGGAGGGCATAACTTGGTCGAG | This work |
| AtLOX2R | TGCGTAGTCTTCTACCGTAATCCG | This work |
| AtJAZ1F | CAGACGTGTAGTCGATTGAGTCAG | This work |
| AtJAZ1R | AAGTTCCATTGACATCAGGCTTGC | This work |
| AtJAZ10F | CAGACGTGTAGTCGATTGAGTCAG | This work |
| AtJAZ10R | AAGTTCCATTGACATCAGGCTTGC | This work |

| **qPCR primers for Arabidopsis genes associated with callose synthesis and defense** | | | |
| --- | --- | --- | --- |
| CYB81F2F | AT5G57220.1 | CATCATCAAAGGGCTCATGCTCAG | This work |
| CYB81F2R |  | AATGTTACGGCCGCAGTATCCG | This work |
| GSL12F | AT5G13000.1 | TTGTCTGCCTGTTGCTGTACCC | This work |
| GSL12R |  | CGACAATAGGCTTGCTGTTATCCC | This work |
| GSL5F | AT4G03550.1 | CCACCACGAGTACATTCAGGTC | This work |
| GSL5R |  | GTACACATCTCGGCTGAGAACC | This work |
| GSL7f | AT1G06490.1 | TCTTCCGCTGTACGCTTTAGGG | This work |
| GSL7R |  | AGCAAGAATTGCCGCCTTAATCTC | This work |
| GT61F | AT3G18170.1 | AACGGTGAATTCCTGCGACGTG | This work |
| GT61R |  | GGCAAGAACACCATGTTCGTCAGC | This work |
| IRX9-LF | AT1G27600.1 | ACAGTGAAGGAGGGTTTCCAGGAG | This work |
| IRX9-LR |  | TCTCGCTTTCATCAGCCACCAC | This work |
| MUR3F | AT2G20370.1 | TTGACTCAATGCTTGCCGGTTG | This work |
| MUR3R |  | TGCCACGTGTATTGCGTGTAGG | This work |
| PEN2F | AT2G44490.1 | TGCGCATAATCCACTTTGGTACG | This work |
| PEN2R |  | AGTCGGATGCTGATGCCAAC | This work |
