# Supplementary material for: Overexpression of a cell wall damage induced transcription factor, OsWRKY42, leads to enhanced callose deposition and tolerance to salt stress but does not enhance tolerance to bacterial infection
Source: BMC Plant Biol. 2018 Sep 3;18:177. doi: 10.1186/s12870-018-1391-5 (PMC6122458; doi:10.1186/s12870-018-1391-5)
Supplement: Supplementary file 10 — Table S2. List of primers. (DOCX 24 kb) [file 12870_2018_1391_MOESM10_ESM.docx]

**Table S3.** **List of bacterial strains and plasmids**

| **Strains/plasmids** | **Relevant characteristics^a^** | **Reference/source** |
| --- | --- | --- |
| **Bacterial Strains** | | |
| *E. coli* DH5α | λ–f80d*lacZ*DM15D(*lacZYA*-*argF*) *U169 recA1 endA hsdR17* (rK– mK–) *supE44 thi-1 gyrA relA1* | Invitrogen |
| BXO43 (*Xoo*) | *rif*-2 ^a^; derivative of wild type Indian isolate (BXO1) | Laboratory collection |
| *Pseudomonas syringae* pv. *tomato* DC3000 | Rif^r^ | Dr. Subhadeep Chatterjee |
| *A. tumefaciens* | | |
| LBA 4404 | TiAch5, pAL4404; T-region, tet^r^, St^r^ | [41] |
| LBA4404/pMDC7/OsWRKY42-2XFLAG | LBA4404/ pMDC7/OsWRKY42; tet^r^, St^r^, Sp^r^, Hygro^r^ | This work |
| LBA4404/pH7FWG2/OsWRKY42 | LBA4404/ pH7FWG2/OsWRKY42; tet^r^, St^r^, Sp^r^, Hygro^r^ | This work |
| **Plasmids** | | |
| pENTR D- TOPO | Kan^r^ | Invitrogen |
| pMDC7 | 17-β-estradiol inducible binary vector, Derived from PER8 vector, Sp^r^, Hyg^r^ | [42] |
| pH7FWG2 | Constitutive overexpression binary vector derived from pPZP200 vector. It has CAMV35S promoter and C-terminal EGFP tag, Sp^r^, Hyg^r^ | Karimi et al., 2002 |
| XVE::OsWRKY42 | 762 bp size fragment of OsWRKY42 gene without stop codon was cloned into pENTR D-TOPO and then recloned into pENTR D-TOPO using a reverse primer containing FLAG tag sequence. The OSWRKY42-2XFLAG was cloned into pMDC7 T-DNA binary vector by performing LR-clonase reaction | This work |
| 35S::OsWRKY42 | 762 bp size fragment without stop codon from pENTR-D TOPO was cloned into pH7FWG2 | This work |

^a^ The *rif*-2 mutation confers resistance to rifampicin; Kan^r^, Hyg^r^, tet^r^ Sp^r^, St^r^ and Rif^r^ indicate resistance to kanamycin, hygromycin, tetracycline, spectinomycin, streptomycin and rifampicin respectively.
